# Correction to “Insights into the roles of long noncoding RNAs in the communication between plants and the environment”

**DOI:** 10.1002/tpg2.70045

**Published:** 2025-05-06

Yuan, C., He, R.‐R., Zhao, W.‐L., Chen, Y.‐Q., & Zhang, Y.‐C. (2023). Insights into the roles of long noncoding RNAs in the communication between plants and the environment. *The Plant Genome*, *16*, e20277. https://doi.org/10.1002/tpg2.20277

Due to an oversight, one of the funding sources that supported the publication fee of this article was not included in the original submission. The National Key Project of Agricultural Science and Technology (Project No. NK20220501) should have been listed in both the Funding and Acknowledgment sections.

We apologize for this error.

